# Multi‐omic expression of the VEGF family relates to Alzheimer's disease across diverse populations

**DOI:** 10.1002/alz.71100

**Published:** 2026-01-28

**Authors:** Julia B. Libby, Kacie D. Deters, Nilüfer Ertekin‐Taner, Minerva M. Carrasquillo, Mariet Allen, Philip De Jager, Vilas Menon, Bin Zhang, Vahram Haroutunian, Allan I. Levey, Nicholas T. Seyfried, Rima Kaddurah‐Daouk, Steve Finkbeiner, Daifeng Wang, Anna K. Greenwood, Abby Vander Linden, Laura Heath, William L. Poehlman, Logan Dumitrescu, Vladislav A. Petyuk, David A. Bennett, Julie A. Schneider, Lisa L. Barnes, Timothy J. Hohman

**Affiliations:** ^1^ Vanderbilt Memory and Alzheimer's Center Vanderbilt University Medical Center Nashville Tennessee USA; ^2^ Department of Integrative Biology and Physiology University of California Los Angeles California USA; ^3^ Department of Neuroscience Mayo Clinic Florida Jacksonville Florida USA; ^4^ Department of Neurology Mayo Clinic Florida Jacksonville Florida USA; ^5^ Department of Neurology Center for Translational and Computational Neuroimmunology Columbia University Medical Center New York New York USA; ^6^ Cell Circuits Program Broad Institute of MIT and Harvard Cambridge Massachusetts USA; ^7^ Mount Sinai Center for Transformative Disease Modeling Icahn School of Medicine at Mount Sinai New York New York USA; ^8^ Departments of Psychiatry and Neuroscience Icahn School of Medicine and JJ Peters VA MIRECC New York New York USA; ^9^ Emory University School of Medicine Atlanta Georgia USA; ^10^ Department of Biochemistry Emory University School of Medicine Atlanta Georgia USA; ^11^ Department of Psychiatry and Behavioral Sciences Duke University Durham North Carolina USA; ^12^ Duke Institute of Brain Sciences Duke University Durham North Carolina USA; ^13^ Department of Medicine Duke University Durham North Carolina USA; ^14^ Center for Systems and Therapeutics Gladstone Institutes San Francisco California USA; ^15^ Department of Physiology University of California San Francisco California USA; ^16^ Department of Neurology University of California San Francisco California USA; ^17^ Waisman Center University of Wisconsin‐Madison Madison Wisconsin USA; ^18^ Departments of Biostatistics and Medical Informatics School of Medicine and Public Health University of Wisconsin‐Madison Madison Wisconsin USA; ^19^ Sage Bionetworks Seattle Washington USA; ^20^ Department of Neurology Vanderbilt Genetics Institute Vanderbilt University Medical Center Nashville Tennessee USA; ^21^ Biological Sciences Division Pacific Northwest National Laboratory Richland Washington USA; ^22^ Rush Alzheimer's Disease Center Rush University Medical Center Chicago Illinois USA

**Keywords:** Alzheimer's disease, brain multi‐omics, bulk RNA sequencing, cognition, diverse populations, neuropathology, TMT‐MS, VEGF

## Abstract

**INTRODUCTION:**

The vascular endothelial growth factor (VEGF) signaling family plays a role in neurodegenerative diseases, including Alzheimer's disease (AD). Previous work has shown widespread effects of the members *FLT1*, *FLT4*, and *VEGFB* on AD outcomes. However, these analyses have focused within the non‐Hispanic White (NHW) population.

**NETHODS:**

The goal of this study was to analyze the effects of the *VEGF* family in underrepresented populations, leveraging large and diverse bulk RNA sequencing and tandem mass tag–mass spectrometry (TMT‐MS) proteomic data. Outcomes included measures of AD pathology and diagnosis.

**RESULTS:**

Within underrepresented populations, we replicated previously reported effects of *FLT1* and *FLT4*, whereby higher protein abundance was observed in the AD brain and was associated with higher neuropathology burden. In stratified analyses, these associations were largely consistent across race and ethnicity.

**DISCUSSION:**

This multi‐omic study on the role of the *VEGF* family in AD emphasizes the need for more representative studies focused on therapeutic targets for AD.

**Highlights:**

- Vascular endothelial growth factor (VEGF) genes and proteins were quantified in four different brain regions. Samples included participants from four different populations.
- Previously observed effects were replicated in diverse populations.
- This study is the largest multi‐omic study of the vascular endothelial growth factor (VEGF) genes among Alzheimer's disease (AD) participants from diverse populations.

## 1 BACKGROUND

The vascular endothelial growth factor (*VEGF*) signaling family is significantly involved in multiple processes related to the growth and maintenance of both vascular cells and neuronal cells.^1^, ^2^ This signaling family includes five ligand encoding genes (*VEGFA*, *VEGFB*, *VEGFC*, *VEGFD*, and placental growth factor [*PGF]*), three receptor genes (*FLT1*, *KDR*, and *FLT4*), and two co‐receptor genes (*NRP1*, *NRP2*). These genes have been implicated in numerous neurological conditions like brain injury,^3^ Parkinson's disease,^4^ stroke,^5^, ^6^ and Alzheimer's disease (AD).^1^, ^7^

Previous work from our group has deeply characterized the effects of the *VEGF* family on AD leveraging multi‐omic approaches.^8^, ^9^, ^10^, ^11^, ^12^ Across our studies, we have provided strong evidence that elevated *VEGFB*, *FLT1*, *FLT4*, and *PGF* in the brain relate to worse cognitive outcomes and higher levels of AD neuropathology.^8^, ^9^, ^10^, ^11^, ^12^ Yet, all of these analyses have been limited to cohorts enriched for non‐Hispanic White (NHW) participants, limiting generalizability in populations that bear a disproportionate risk for AD and related dementias.^13^, ^14^ The Accelerating Medicine Partnership for Alzheimer's Disease (AMP‐AD) Diversity Initiative has provided an exciting opportunity to expand our work to groups underrepresented in science and clarify which *VEGF* family associations are observed across populations.

Therefore, the goal of this study is to investigate whether previously observed effects of the *VEGF* family on AD replicate within more diverse populations using data from cohorts dedicated to this goal. Specifically, we investigate the effects of the various *VEGF* family members on measures of AD pathology and AD diagnosis leveraging data generated from multiple brain regions at both the transcriptome and proteomic level. Finally, we formally assess the effects within underrepresented populations to examine if previously observed effects are generalizable across populations.

## 2 METHODS

### 2.1 Participants

Data from *post mortem* brain samples were obtained from the AMP‐AD Diversity Initiative, which collected data enriched for donors from African American (AA) and Latin American (LA) populations within the United States from five AMP‐AD contributing institutions, including Mayo Clinic, Rush University Medical Center, Mount Sinai, Columbia University, and Emory University. Further population information can be found in greater detail within the AMP‐AD cohort study, which breaks down population demographics by contributing institution.^15^ In order to reach the sample sizes required for the AMP‐AD Diversity Initiative, brains from multiple studies and brain banks were ascertained from each of the contributing universities. Brains from the Mayo Clinic came from the Mayo Clinic Brain Bank, the Banner Sun Health Research Institute Brain Bank, or from the University of Florida Brain Bank. The brains from Mt. Sinai came from the Mt. Sinai Brain Bank. The brains from Rush University came from the Clinical Core of the Rush AD Research Center (ADRC), the Latino Core Study, the Minority Aging Research Study, the Religious Orders Study, or the Rush Memory an Aging Project. The brains from Columbia University were ascertained from the Bigg Institute Brain Bank, the Estudia Familiar de Influencia Genetica en Alzheimer Study, the National Institute on Aging AD Family Based Study, the Washington Heights Inwood Columbia Aging Project, or the Columbia ADRC. Each of these cohort studies and brain banks differed in their acquisition protocols as outlined in the flagship manuscript for AMP‐AD Diversity Initiative.

**RESEARCH IN CONTEXT**

1. **Systematic review**: The authors reviewed the literature using traditional sources (e.g., PubMed, Google Scholar). Many studies have characterized the role of the vascular endothelial growth factor (VEGF) family in Alzheimer's disease (AD) within the non‐Hispanic White (NHW) population, though few to date have looked within diverse populations. A representative summary of discovery multi‐omic analyses of *VEGF* within AD, including an emphasis on the largest studies to date and the lack of diversity within these studies, were appropriately cited.
2. **Interpretation**: This study replicated multiple effects within diverse populations of *VEGF* gene expression on AD neuropathology and cognitive performance, previously recorded primarily in the NHW population.
3. **Future directions**: Replication of the results reported here is needed, specifically in larger and more diverse cohorts. Additionally, future work is needed to understand the role of social determinants of health on population effects.

### 2.2 Phenotype data

Sample collection methods and neuropathological categorization decisions varied across contributing sites. Therefore, the AMP‐AD team harmonized the data across sites following the protocols established by Alzheimer's Disease Sequencing Project Phenotype Harmonization Consortium (ADSP‐PHC; https://vmacdata.org/adsp‐phc). The phenotype data collected from these sites were harmonized by mapping onto common data elements that were largely based on the NACC neuropathology data dictionary. Resulting phenotypes included categorical measures of brain amyloidosis (Thal phasing, Consortium to Establish a Registry for Alzheimer's Disease [CERAD] staging), and tau tangle pathology (Braak staging). Similarly, clinical diagnostic methods largely varied across contributing sites with very few brain banks providing the clinical data needed to make a clinical diagnosis. Therefore, AMP‐AD made the decision to focus on a neuropathology‐derived diagnosis. Using CERAD and BRAAK staging, participants were placed into neuropathology diagnostic groups of either “Control”, “AD”, “Other”, or “Missing/Unknown”. A “Control” diagnosis was assigned to participants with BRAAK stage ≤ III and CERAD measure equal to none or sparse neuritic plaques. An “AD” diagnosis was assigned to participants with BRAAK stage ≥ IV and CERAD measure equal to moderate or frequent neuritic plaques. Any participants not falling in either of these criteria were assigned to “Other”, while participants without comprehensive neuropathology data were assigned “Missing/Unknown”. Further categorization decisions made by AMP‐AD have been previously characterized in greater detail.^15^

For race‐stratified models, population variables were combined to create a variable reflecting both reported race and ethnicity, while maximizing sample sizes for the groups. The groups created included NHW, non‐Hispanic Black (NHB), Hispanic, and Other for the few participants that didn't fall into the previous categories. Due to a low sample size, the individuals that fell into the “Other” category were not included in race‐stratified analyses.

### 2.3 Bulk RNA sequencing

Bulk RNA sequencing was performed across the contributing centers within four brain regions: dorsolateral prefrontal cortex (DLPFC), caudate nucleus (CN), superior temporal gyrus (STG), and temporal pole (TP). Data are available on the AMP‐AD Knowledge Portal (syn2580853). Brain regions were selected to capture molecular profile differences across regions at varying stages of AD neuropathology. Processing and alignment protocol followed published protocol.^16^ To summarize, reads were assigned to Ensembl (zGRChr38, v112) reference genome using STAR (v2.5.2b). From aligned reads, gene counts computed using featureCounts from Subread (v2.0.0), and Picard metrics were calculated (v2.18.27). Sample filters included removing samples with RNA integrity number (RIN) < 3, *post mortem* interval (PMI) > 24 h, outliers in principle component analysis (PCA), or samples who were missing RIN or any required demographic variables. Gene filters included removing genes with missing gene length or guanine–cytosine (GC) content prior to normalization. Remaining gene counts were quantile‐normalized and then filtered so that any expression values > 3 standard deviations (SD) from the mean were excluded.

To prevent observed effects from being driven by technical differences, expression went through further iterative normalization using software from the limma R package,^17^ adjusting for batch, age at death, RNA integrity number (RIN), and percentage of coding, intronic, and intergenic bases, and sensitivity models were rerun with this expression data.

### 2.4 Tandem mass tag–mass spectrometry base proteomics

Protein abundance was quantified using isobaric tandem mass tag–mass spectrometry (TMT‐MS) at Emory University on 1,105 DLPFC tissue samples and 280 STG tissue samples from contributing centers (syn53185479) using protocol previously described.^15^ Quality control of resulting expression included removing proteins that have missing data in > 50% of the samples, taking the ratio of the protein abundance to all protein abundance for that sample to account for sample loading differences, log2 transformation of the ratio, removing samples that were outliers in PCA analysis, and regressing out batch effects. The resulting data included 1,086 DLPFC samples and 278 STG samples. Five STG samples missing phenotype data were excluded in analyses, leaving 273 STG samples. Quantification produced measurements for FLT1, NRP1, and NRP2 in DLPFC samples and FLT1, FLT4, NRP1, and NRP2 in STG samples.

### 2.5 Statistical analyses

Statistical analyses were completed using R (version 4.3.0) and code is available from the authors upon request. Significance was set at a priori to *α* = 0.05. Analyses in this study were run in all tissues, while excluding the TP region due to insufficient data for regressions.

Generalized linear regression models were used to test *VEGF* associations with binary variables including the presence or absence of amyloid pathology and diagnosis. For the model looking at diagnosis, we removed participants with a diagnosis of “Other” or “Missing/Unknown”, leaving diagnostic groups of “Control” and “AD”. Ordinal logistic regression models were used to test associations with ordinal variables including Thal phase, Braak stage, and CERAD score. Additional race‐stratified models looking within NHW, NHB, and Hispanic populations separately were used to test *VEGF* associations with pathology and diagnosis within these specific populations. All models covaried for sex, apolipoprotein E (*APOE)* ‐ε4 carrier status, age at death, and PMI.

All nominally significant associations, classified as an uncorrected *p* < 0.05, are reported in the text. *p*‐values were corrected for multiple comparisons for all VEGF predictors across all data types and outcomes using false discovery rate (FDR) procedure, including 615 models. All results are included in Table S1 with both uncorrected and corrected p‐values. Throughout the text, nominal p‐values that passed FDR correction are denoted by an asterisk.

## 3 RESULTS

Participant characteristics are presented by data type and tissue in Table 1. Bulk RNA sequencing data included a higher percentage of NHB and Hispanic participants than NHW, while TMT‐MS data included a higher percentage of NHW participants than NHB, followed by Hispanic. Both males and females were well‐represented across tissues and data types. Most participants were cognitively impaired, with a little less than half being *APOE*‐ε4 carriers.

**TABLE 1:** Participant characteristics

Parameter	Bulk RNA			TMT	
	DLPFC (*N* = 508)	STG (*N* = 498)	CN (*N* = 449)	DLPFC (*N* = 980)	STG (*N* = 238)
Female, *N* (%)	274 (54)	265 (53)	260 (58)	579 (59)	121 (51)
Non‐Hispanic Black, *N* (%)	179 (35)	147 (30)	179 (40)	271 (28)	76 (32)
Hispanic, *N* (%)	208 (41)	205 (41)	235 (53)	259 (27)	48 (20)
Non‐Hispanic White, *N* (%)	108 (21)	140 (28)	26 (6)	430 (44)	114 (48)
Age at death	78.5 ± 11.5	78.2 ± 11.5	78.7 ± 11.2	80.5 ± 10.6	76.3 ± 12.9
*Post mortem* interval	8.9 ± 5.6	8.6 ± 5.5	9.6 ± 5.8	10.4 ± 13.7	11.0 ± 10.4
Cognitively normal, *N* (%)	84 (17)	112 (23)	55 (12)	213 (22)	63 (27)
*APOE4* carriers, *N* (%)	214 (43)	185 (38)	191 (45)	374 (41)	112 (48)

Abbreviations: APOE, apolipoprotein E; CN, caudate nucleus; DLPFC, dorsolateral prefrontal cortex; STG, superior temporal gyrus; TMT, tandem mass tag.

### 3.1 VEGF inter‐tissue correlations

To aid in interpretability of analyses performed on the same genes across various brain tissues, we first examined the correlation of bulk RNA expression of each *VEGF* gene in the three brain tissues in overlapping samples (*N* = 312). Most of the *VEGF* genes were moderately positively correlated across tissues (*r* > 0.25). However, *FLT4* expression in the CN was weakly correlated with expression in the STG (*r* = 0.08) and the DLPFC (*r* = 0.1). Additionally, *NRP2* expression in the CN was slightly less correlated with STG expression than the average across genes (*r* = 0.19).

### 3.2 VEGF associations with measures of AD pathology and AD diagnosis

#### 3.2.1 Results across racial and ethnic groups

**FIGURE 1:**
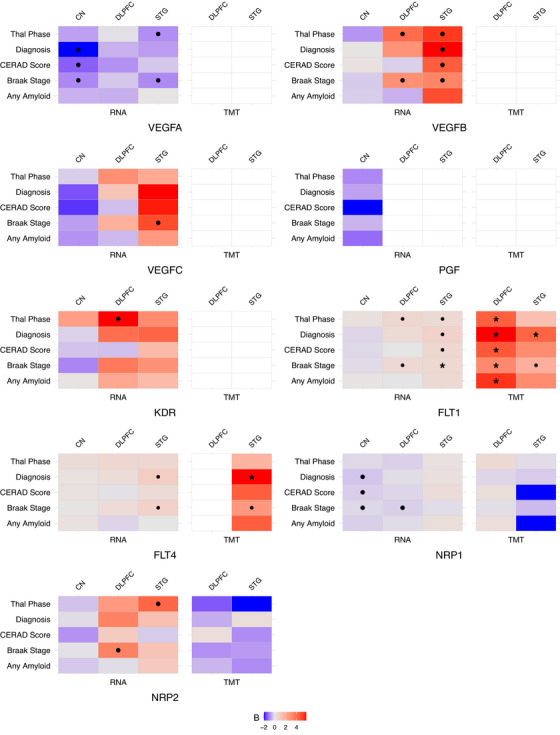
This figure illustrates associations of *VEGF* family members and outcomes across the two datatypes, while also indicating which associations passed corrections for all tests. The *x*‐axis for each gene is the data types, with the type of data measurement on the bottom and brain region on the top. The *y*‐axis contains the outcomes. The color of each square indicates the strength of the association where more red colors indicate positive associations, and more blue colors indicate negative associations. A gray colored box indicates a beta closer to zero. White boxes indicate no measured expression for the given gene. Associations reaching nominal significance are indicated by a black dot, and associations passing corrections for all tests are indicated with a black asterisk. Tissue CN, caudate nucleus; DLPFC, dorsolateral prefrontal cortex; STG, superior temporal gyrus; VEGF, vascular endothelial growth factor

Figure 1 summarizes results for *VEGF* gene associations, with all racial and ethnic groups combined, with AD pathology measures and diagnosis for all tissues and data types. Full analysis results may be found in Table S1. At the transcript level, increased *VEGFA* expression was associated with lower amyloid and tau including CERAD stage in the CN (*β* = −0.55, *p* = 0.002), Braak stage in the CN (*β* = −0.38, *p* = 0.005) and STG (*β* = −0.37, *p* = 0.006), and Thal phase in the STG (*β* = −0.35, *p* = 0.04). Increased *VEGFB* expression was associated with higher measures of pathology including a higher Thal phase in the STG (*β* = 1.09, *p* = 0.005) and the DLPFC (*β* = 0.84, *p* = 0.02), higher Braak stage in the STG (*β* = 0.69, *p* = 0.01) and in the DLPFC (*β* = 0.61, *p* = 0.03), and a higher CERAD score in the STG (*β* = 0.93, *p* = 0.02). Within the STG, *VEGFC* was associated with a higher Braak stage (*β* = 0.37, *p* = 0.02), and increased *KDR* expression within the DLPFC was associated with a higher Thal phase (*β* = 0.39, *p* = 0.04).

Widespread effects of *FLT1* expression in the brain were observed on measures of pathology. Within the DLPFC, increased expression of *FLT1* was associated with a higher Braak stage (*β* = 0.45, *p* = 0.003) and Thal phase (*β* = 0.54, *p* = 0.004). In the STG, expression of *FLT1* was associated with a higher Braak stage (*β* = 0.48, *p* = 0.002), Thal phase (*β* = 0.43, *p* = 0.03), and CERAD score (*β* = 0.50, *p* = 0.04). Increased expression of *FLT4* was also associated with a higher Braak stage in the STG (*β* = 0.45, *p* = 0.01). *NRP2* expression in the STG was associated with a higher Thal phase (*β* = 0.69, *p* = 0.03). Interestingly, *NRP1* expression was associated with a lower Braak stage in the DLPFC (*β* = ‐0.56, *p* = 0.02) and a lower CERAD score in the CN (*β* = −0.60, *p* = 0.03).

**FIGURE 2:**
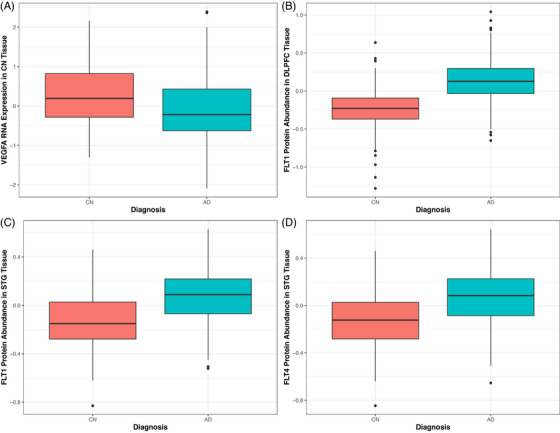
(A) VEGFA RNA expression association with diagnosis in the CN. (B, C) FLT1 protein expression associations with diagnosis in the DLPFC and STG, respectively. (D) FLT4 STG protein expression with diagnosis. Diagnosis coding: CN, cognitively normal; AD, Alzheimer's disease. DLPFC, dorsolateral prefrontal cortex; VEGFA, vascular endothelial growth factor A

When looking at transcriptomic associations with AD diagnosis, multiple genes were upregulated in the AD brain including *VEGFB* (*β* = 1.21, *p* = 0.007), *FLT1* (*β* = 0.68, *p* = 0.007), and *FLT4* (*β* = 0.55, *p* = 0.04) in the STG, whereas *VEGFA* expression was downregulated in the CN (Figure 2A, *β* = −0.83, *p* = 0.0009*).

**FIGURE 3:**
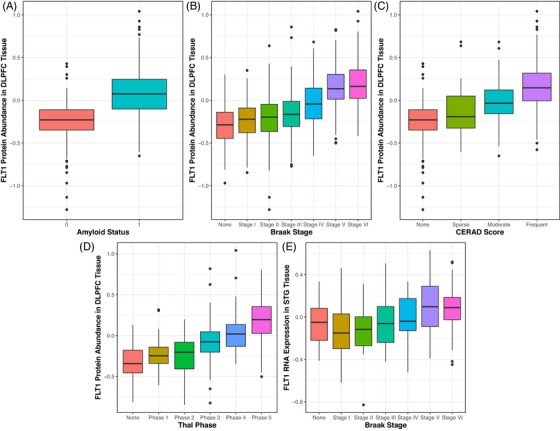
(A–D) FLT1 DLPFC protein expression associations with amyloid status, Braak stage, CERAD score, and Thal phase, respectively. (E) FLT1 STG RNA expression association with Braak stage. CERAD, Consortium to Establish a Registry for Alzheimer's Disease; DLPFC, dorsolateral prefrontal cortex; STG, superior temporal gyrus

At the proteomic level, widespread effects of FLT1 expression were also observed across tissues. Within the DLPFC, FLT1 expression was associated with the presence of amyloid (Figure 3A, *β* = 5.33, *p* = 1.48e^−19^*), a higher Braak stage (Figure 3B, *β* = 3.17, *p* = 7.13e^−32^*), CERAD score (Figure 3C, *β* = 4.58, *p* = 1.18e^−29^*), and Thal phase (Figure 3D, *β* = 4.38, *p* = 3.34e^−20^*). Within the STG, higher expression of FLT1 was associated with a higher Braak stage (Figure 3E, *β* = 2.05, *p* = 0.001). Additionally, increased expression of FLT4 in the STG was associated with a higher Braak stage (*β* = 1.68, *p* = 0.006).

Proteomic effects on AD diagnosis included upregulation of FLT1 in the DLPFC (Figure 2B, *β* = 5.80, *p* = 1.19 × 10^−24^*) and STG (Figure 2C, *β* = 4.26, *p* = 5.67 × 10^−05^*) and upregulation of FLT4 in the STG region (Figure 2D, *β* = 3.64, *p* = 0.0002*).

#### 3.2.2 Effects by race/ethnicity

**FIGURE 4:**
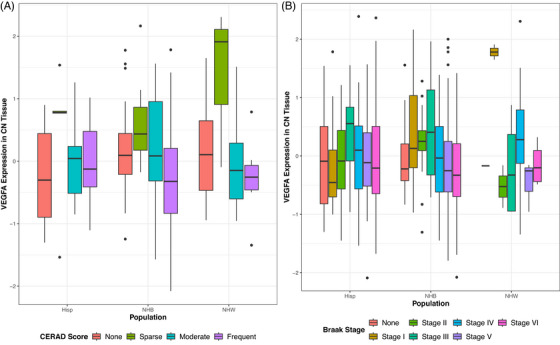
Associations of *VEGFA* RNA expression in the CN with (A) CERAD score and (B) Braak stage within each population. CERAD, Consortium to Establish a Registry for Alzheimer's Disease; CN, caudate nucleus; VEGFA, vascular endothelial growth factor A

When looking at effects within each population separately, multiple strong signals within NHB and Hispanic populations that corrected for multiple comparisons were observed that were previously observed solely in NHW. At the transcriptomic level, within the NHB population, *VEGFA* expression in the CN was associated with lower CERAD scores (Figure 4A, *β* = −1.03, *p* = 8.55 × 10^−05^*) and Braak stages (Figure 4B, *β* = −0.74, *p* = 0.0002*).

**FIGURE 5:**
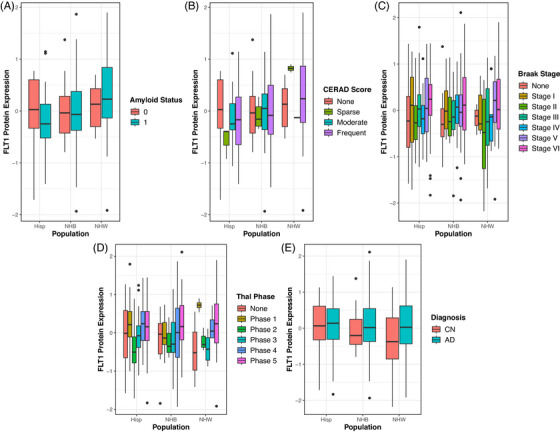
Associations of FLT1 protein expression in the DLPFC with (A) amyloid status, (B) CERAD score, (C) Braak stage, (D) Thal phase, and (E) diagnosis within each population. Diagnosis coding: CN, cognitively normal; AD, Alzheimer's disease. CERAD, Consortium to Establish a Registry for Alzheimer's Disease; DLPFC, dorsolateral prefrontal cortex

At the proteome level, widespread effects of FLT1 expression within the DLPFC was observed within the NHB and Hispanic populations. Within the NHB population, FLT1 protein expression in the DLPFC was associated with worse measurements of pathology, including the presence of amyloid pathology (Figure 5A, *β* = 3.94, *p* = 3.63 × 10^−05^*), a worse CERAD score (Figure 5B, *β* = 5.53, *p* = 2.00 × 10^−09^*), Braak stage (Figure 5C, *β* = 2.84, *p* = 1.74 × 10^−08^*), and Thal phase (Figure 5D, *β* = 3.97, *p* = 4.29 × 10^−09^*). Similar effects were observed within the Hispanic population where higher FLT1 protein expression in the DLPFC was associated with a higher Braak stage (Figure 5C, *β* = 3.23, *p* = 4.80 × 10^−08^*), Thal phase (Figure 5D, *β* = 4.35, *p* = 8.07 × 10^−07^*), and CERAD score (Figure 5B, *β* = 3.82, *p* = 0.001*). Additionally, DLPFC expression of FLT1 was associated with AD cases in both NHB (Figure 5E, *β* = 5.10, *p* = 2.90 × 10^−06^*) and Hispanic (Figure 5E, *β* = 4.36, *p* = 0.001*) populations.

All nominally significant stratified results are included in Table S1.

## 4 DISCUSSION

In this analysis, we replicated previously reported effects of multiple members of the VEGF gene family in a highly diverse population including NHW, NHB, and Hispanic populations. Specifically, we observed robust effects of *FLT1* expression at both the transcript and protein level on multiple measures of AD pathology and diagnosis. Also consistent with previous findings, we observed protective effects of *VEGFA* and *NRP1* expression, and detrimental effects of *VEGFB*, *KDR*, *NRP2*, and *FLT4* expression on AD.

*FLT1* expression was higher among AD cases compared to controls and higher expression was associated with higher levels of AD neuropathology. These effects have been observed by our group multiple times across varying tissues, cohorts, and now across diverse populations. We first observed the effect of *FLT1* expression on cognitive trajectories and AD diagnosis in the prefrontal cortex (PFC).^9^ This signal was also comprehensively observed in a separate publication on multiple levels including bulk RNA sequencing, TMT, and single‐nucleus RNA sequencing.^11^ Specifically, *FLT1* expression in the DLPFC both at the transcriptomic and proteomic levels was associated with higher levels of AD pathology, including amyloid and tau, and was associated with worse cognitive performance across multiple brain tissues. When looking at cell‐specific effects, *FLT1* expression in microglial cells appeared to be driving the effect on cognition, which was later replicated in an independent single‐nucleus dataset.^12^ In this analysis, we observed our strongest effects on pathology and diagnosis at the proteomic level, further solidifying a potential mechanistic role of *FLT1* expression on AD that is consistent across populations.

We also observed detrimental effects of *VEGFB* expression on pathology levels and diagnosis across the DLPFC and STG brain tissues. While these effects didn't correct for multiple comparisons, these results have been observed consistently across studies.^9^, ^11^, ^12^ The appearance of these signals in addition to *FLT1* indicate a strong role of the *FLT1*‐*VEGFB* signaling pathway in neurodegeneration and neuronal dysfunction. *VEGFB* has been shown to play a role in neuronal survival and regeneration.^18^, ^19^, ^20^ While these effects are beneficial under normal conditions, in the presence of AD pathology, *VEGFB* signaling has been shown to result in an increase in angiogenesis and immune activation.^21^ However, the role of *VEGFB* signaling within disease conditions may be cell type specific. Notably, in our small nuclear RNA (snRNA) results reported earlier this year,^12^ we identified a differential association for *VEGFB* by cell type, where higher levels were associated with worse outcomes in oligodendrocytes, but better outcomes in neurons. Given that the present data are tissue homogenate, it is possible that differences in cell fraction contribute to the heterogeneity observed across various studies. Future single‐cell analysis could help clarify these differences.

In addition to the *FLT1* and *VEGFB* signals, multiple additional signals were observed that were consistent with previous findings. In particular, a signal that has continued to appear across studies is the detrimental role of *FLT4* on AD pathology and diagnosis. Here we observed a strong association between increased FLT4 expression at the proteomic level and AD diagnosis, surviving correction for multiple comparisons. While the direction of this effect has varied across brain regions,^11^ *FLT4* appears to play a strong role in AD related pathways. Specifically, *FLT4* signals have consistently appeared alongside *FLT1* signals, indicating common pathways may be at play driving the upregulation of both *FLT1* and *FLT4* most likely in response to the presence of AD pathology^22^, ^23^.

We also observed significant and nominal effects of the receptor and ligand pair *VEGFA* and *KDR*,^1^ respectively, where increased *VEGFA* expression across regions was protective against AD pathology, and *KDR* expression in the DLPFC was associated with worse AD pathology. These weaker signals have shown up less consistently across studies,^8^, ^9^ but indicate a potential role of related pathways involving this pair, including the canonical angiogenesis pathway which is important for the production and healing of blood vessels.^24^ We also observed nominal effects of *NRP1* and *NRP2*, where *NRP1* appeared protective against AD pathology and *NRP2* associated with worse AD pathology. These signals rarely appear across studies, but seem to have tissue and cell specific effects when appearing,^10^, ^11^, ^12^ suggesting more work is needed to clarify these complex associations.

Due to the heterogeneous nature of AD across different populations, we performed sensitivity analyses looking at the effects within underrepresented groups. The observed results were consistent within these populations with previously observed associations reported in NHW populations. These encouraging results emphasize the potential of VEGF therapeutic targets in the general population, specifically *FLT1* which had widespread detrimental effects on pathology and diagnosis across populations. However, one study alone is not enough evidence to validate this effect across all populations. There is still a great need for diversity in AD studies, particularly at the clinical trial level. Diverse populations have been historically, and continue to be, left out or underrepresented in AD trials. One report in 2022 estimated across 101 AD drug clinical trials, on average 94% of participants were White individuals.^25^ With NHB and Hispanic populations being at a greater risk for AD,^14^ the inclusion of these populations in studies is greatly needed to truly understand and safely treat AD in the general population.

This study has multiple strengths. It is the largest and most diverse multi‐omic analysis of the VEGF signaling family effects on AD and aging to date, leveraging harmonized data from the AMP‐AD Diversity Initiative. In addition, this study evaluated VEGF family associations across multiple brain regions at the transcript and protein level. Despite the extensive multi‐omic data used, we were limited to looking within only a few brain regions for the bulk RNA data and even fewer for the TMT proteomic data. More regional expression data are needed to better fully characterize the effect *VEGF* has in the AD and aging brain. Another limitation was the lack of continuous measurements of pathology or cognition that may have reduced our statistical power relative to previous studies. Additionally, due to the heterogeneous nature of the data collected by each contributing site, we were limited to looking at a neuropathology‐derived diagnosis without a clinically derived diagnosis for validation. Additional studies specifically looking at a clinical diagnosis are needed for validation of our observed effects on a neuropathology‐derived diagnosis. Further, while the study here is diverse, we had a limited set of covariates that could fully evaluate social determinants of health, creating challenges in the interpretation of population effects.

To conclude, we provide robust evidence for the detrimental role of *FLT1* signaling in AD across populations and build on our previous work in NHW populations to clarify the role of the VEGF family in the AD brain in populations historically underrepresented in science. Future studies characterizing the brain at single‐cell resolution within diverse populations, and the modifying role of gene (e.g., *APOE*) and genetic ancestry effects will help move these novel candidates forward as potential targets for precision interventions.

## CONFLICT OF INTEREST STATEMENT

R.K.D. is an inventor on a series of patents on use of metabolomics for the diagnosis and treatment of CNS diseases and holds equity in Metabolon Inc., Chymia LLC and PsyProtix. T.J.H. is on the scientific advisory board for Vivid Genomics, is deputy editor for Alzheimer's & Dementia: Translational Research and Clinical Intervention, and is Senior Associate Editor for Alzheimer's & Dementia. J.B.L., K.D.D., N.E.T., M.M.C., M.A., P.D.J., S.F., D.W., A.K.G., A.V.L., L.H., W.L.P., L.D., V.A.P., D.A.B., J.A.S., and L.L.B. have nothing to disclose. Author disclosures are available in the Supporting Information

## CONSENT STATEMENT

Studies included in the AMP‐AD Diverse Cohort Study were approved by the institutional review board (IRB) at the respective institutions, including the Mayo Clinic, Mount Sinai, Rush University Medical Center, Emory University, and Columbia University. All human subjects provided informed consent and signed an Anatomic Gift Act. Secondary analyses of this extant data were approved by the Vanderbilt University Medical Center IRB. All Research was conducted according to the ethical standards outlined in the Declaration of Helsinki.

## Supporting information



Supporting Information

Supporting Information
